# An unusual case of perforation of the alimentary canal following Bigu: A Taoist fasting technique

**DOI:** 10.1097/MD.0000000000008653

**Published:** 2017-12-01

**Authors:** Jun Wang, Jun Jin, Xiaofeng Xue, Yan Hao, Dongrong Li, Shan Xu, Fang Huang

**Affiliations:** a Department of Intensive Care Medicine; b Department of General Surgery, The First Affiliated Hospital of Soochow University, Suzhou, Jiangsu, China.

**Keywords:** Bigu, perforation of alimentary canal, Taoist medicine

## Abstract

**Rationale:**

Bigu is a Taoist fasting technique interpreted as avoiding grains in the Encyclopedia of China. This technique has been used from ancient times to the present day in China and other parts of the world to achieve good health, weight loss, longevity, and even immortality. A variety of health problems have been identified in relation to the severe diet during Bigu. However, perforation of the alimentary canal has not been reported to be associated with Bigu. In the present study, we illustrated an unusual case of perforation of the alimentary canal in relation to Bigu.

**Patient concerns:**

A 36-year-old woman was admitted to our hospital after falling into a coma. One month before admission, she had black stool accompanied by dizziness and fatigue, while the symptoms progressively worsened. The patient reported that she stopped the intake of meat for 5 years, and further practiced Bigu for 5 months, eating only fruits and vegetables, and avoiding grains and meat.

**Diagnosis:**

Preformation of the alimentary canal.

**Interventions:**

Gastric bypass operation, also known as Roux-en-Y anastomosis, was undertaken. Since the patient developed thrombus with edema on the right upper limb after surgical intervention, she was subsequently treated with anticoagulation therapy using low-molecular weight heparin.

**Outcomes:**

The patient's symptoms were remarkably improved and exhibited signs of recovery in follow-up examinations.

**Lessons:**

The case has raises serious concerns about practicing Bigu. Furthermore, it is strongly advocated that a state of Bigu for a long period of time can even be dangerous.

## 1 Introduction

Bigu, which was literally interpreted as avoiding grains or abstaining from 5 cereals in the Encyclopedia of China, is a Taoist fasting technique developed in context of Taoist philosophy and has been used in ancient China to seek longevity and even immortality.^[1–3]^ Generally, in a state of Bigu, an individual lives on air alone for nourishment, entailing absolutely no food or water intake, or abstaining from grains and meat, significantly cutting off the supplement of food to less than the minimum required to maintain normal activities, and living on water, vegetables, fruits, nuts, and Chinese herbal medicines.^[1–4]^ At present, a large number of individuals in China and other parts of the world continue to practice Bigu with a strong belief that weight loss, health, longevity, and immortality could be achieved through this approach. In fact, people who undergo short-term fasting may gain certain benefits. Furthermore, it has been supported by studies on the effects of caloric restriction on physiological parameters, such as the longevity demonstrated when adhering to a calorically restricted diet, which led to better health and a longer lifespan, when compared with a control group that adhered to a self-regulated diet.^[5]^ However, Taoist authors that reported on the severe diet method of Bigu with no intake of grains and meat revealed a wide range of health problems, including weakness, difficulties in moving, sleepiness, constipation, diarrhea, etc. To date, perforation of the alimentary canal in relation to Bigu has not been reported.

In the present case report, we depicted perforation of the alimentary canal in a Chinese woman who adhered to the Taoist fasting technique known as Bigu for a period of time by eating vegetables and fruits alone, without taking any grains and meat. To the best of our knowledge, this is the first case with perforation of the alimentary canal observed in a patient who practices the Taoist fasting technique Bigu. This case provides a unique opportunity to share the characteristics of the clinical and laboratory tests, the care undertaken to treat these symptoms, and clinical outcomes. In addition, this may advance our understanding of the adverse effect of Bigu, and improve the care for similar patients in the future.

## 2 Case presentation

The study was approved by the Ethics Committee of The First Affiliated Hospital of Soochow University. Informed consent was obtained from the individual participant included in the study.

### 2.1 Patient description

A 36-year-old woman was admitted to our hospital after falling into a coma. One month before hospital admission, she started to have black stool accompanied by dizziness and fatigue, which were left untreated. The symptoms worsened 5 days before hospitalization, including an increase in frequency of black stool, abdominal pain, and drowsiness. The patient was reported to be generally healthy with no history of chronic diseases. However, she has been a vegetarian, stopped the intake of meat for 5 years, and further practiced the Taoist fasting technique known as Bigu for 5 months. She only ate raw fresh fruits and vegetables, and avoided any type of grains and meat before she became ill.

### 2.2 Clinical examination and diagnosis

**Figure 1:**
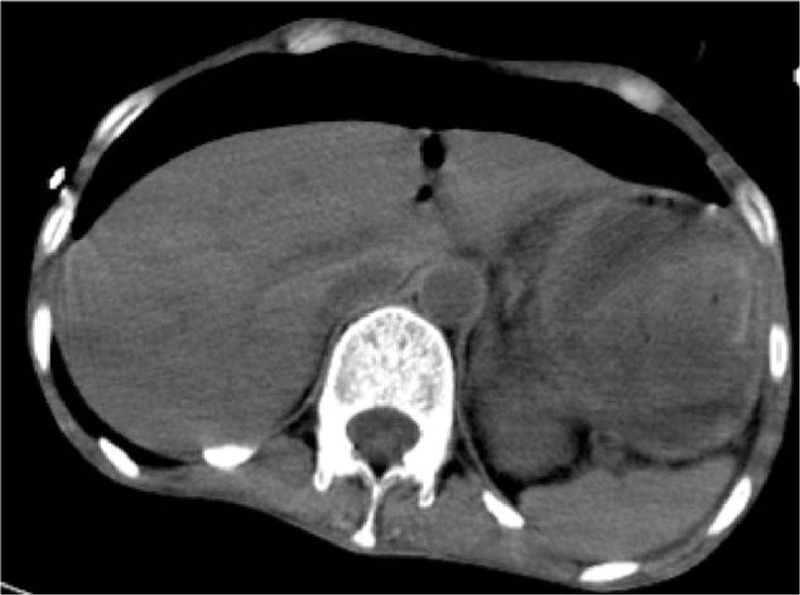
Enhanced computed tomography (CT) imaging of the abdomen. The patient underwent abdominal CT scanning at the time of hospitalization, and a large amount of gas within the peritoneal cavity was detected, which is known as pneumoperitoneum (PM).

At the time of hospital admission, she presented unconsciousness with difficulty in breathing. Physical examination revealed the following: blood pressure, 81/37 mm Hg; heart rate, 140 to 150 BPM; body mass index (BMI), 16.9; undetectable oxygen on a pulse oximeter device. Laboratory tests indicated the following: total white blood count (WBC), 11.38 × 10^9^/L; hemoglobin (HB), 13 g/L; platelet (PLT) count, 188 × 10^9^/L; hypersensitive C-reactive protein (hs-CRP), 146 mg/L; total bilirubin (TBIL), 21.6 μmol/L; alanine aminotransferase (ALT), 269 U/L; aspirate aminotransferase (AST), 360 U/L; creatinine (Cr), 189 μmol/L; blood urea nitrogen (BUN), 14.39 mmol/L; albumin (Alb), 20.9 g/L; pH value, 6.96; pressure of carbon dioxide (PaCO_2_), 15 mm Hg; undetectable pressure of oxygen, HCO_3_^−^, 4.7 mmol/L; lactic acid (Lac), 22 mmol/L; prothrombin time (PT), 22.1 seconds; activated partial thromboplastin time (APTT), 113.9 seconds; fibrinogen (Fbg), 1.0 g/L. Furthermore, abdominal enhanced computed tomography (CT) scanning revealed large amounts of pneumoperitoneum (Fig. 1). According to these clinical examinations and diagnosis criteria, the patient was diagnosed with preformation of the alimentary canal. The severity of the disease was evaluated using the acute physiology and chronic health evaluation II (APCHE II) scale, which resulted to an integer score of 37, and the sequential organ failure assessment (SOFA) scale, which revealed a score of 8.

### 2.3 Treatment, outcome, and follow-up

**Figure 2:**
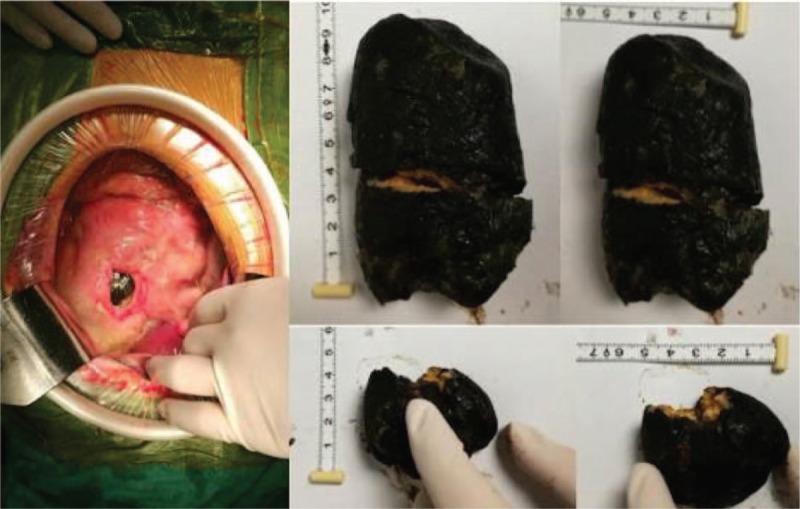
Perforation and lithiasis in the stomach were revealed during an operational procedure. The perforation was located on the lesser curvature, which was 5 cm × 4 cm in size.

**Figure 3:**
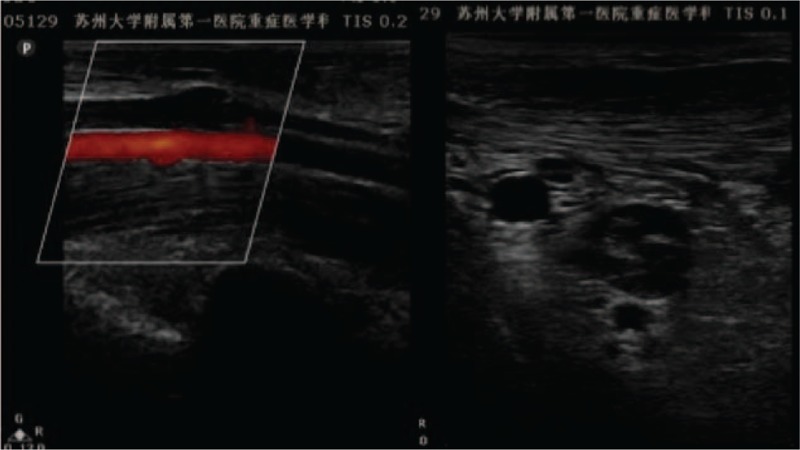
Thrombus within the right of the humeral vein, axillary vein, and subclavian vein are displayed.

Immediately after the patient was admitted to the intensive care unit (ICU), tracheal intubation with mechanical ventilation were performed to reduce the blockage of the airway caused by the coma, and treatment against shock with fluid infusion (LR) of human Alb, fresh plasma, and red blood cells (RBCs) were performed, followed by the maintenance of blood pressure with noradrenaline and antiinfective treatment. The next day following the treatment, the patient revealed an increase in HB of 80 g/L, while total WBC decreased to 1.93 × 10^9^/L without significant improvement in coagulation. The patient received surgical intervention, during which a huge perforation with size of 5 cm × 4 cm on the lesser curvature and a huge lithiasis in the stomach complicated by infection in the abdomen were revealed. Hence, gastric bypass operation, which is well-known as Roux-en-Y anastomosis, was performed (Fig. 2). At 1 day postoperation, a decrease in Lac level (3.8 mmol/L) was observed. Three days later, the patient started enteral nutrition via a nasointestinal feeding tube in combination with parenteral nutrition. At 11 days postoperation, noradrenaline was stopped. Caspofungin was administrated after *Candida albicans* was detected in the blood and ascites of the patient. The patient also developed thrombus within the right of the humeral vein, axillary vein, and subclavian vein (Fig. 3), which were accompanied by edema on the right upper limb. However, computed tomographic pulmonary angiography (CTPA) scans indicated no thrombus in the pulmonary vessels. The patient was treated with anticoagulation therapy using low-molecular weight heparin. Unfortunately, the patient developed ICU-acquired weakness (ICUAW), which manifested as myasthenia and difficulty in weaning. After 40 days of mechanical ventilation and bedside rehabilitation, the patient gradually recovered. BMI increased from a lowest value of 11.7 to 15.8. Furthermore, the patient was able to continuously climb 10 floors, and was discharged. During a follow-up examination, no fistula of the intestine was detected in a contrast examination of the alimentary tract, as well as in other laboratory and clinical tests performed. The patient exhibited signs of recovery.

## 3 Discussion

In the present case, to our knowledge, this is the first time that an unusual perforation of the alimentary canal along with other symptoms were described in a Chinese woman who practiced Bigu for a period of time, and lived on vegetables and fruits as the only source of nutrition. After the patient's clinical conditions were effectively treated, the patient was discharge and exhibited a satisfactory prognosis in the follow-up examination.

To date, perforation of the alimentary canal related to Bigu fasting in Taoist medicine is uncommon. In the present case, we detected a huge perforation of 5 × 4 cm in size on the lesser curvature, accompanied by a huge lithiasis, which was likely to result from her long-term consumption of raw vegetables and fruits. It is undoubted that fresh vegetables and fruits are an important part of our balanced and healthy diet, and compelling scientific evidence has demonstrated that we gain a great number of benefits from a diet rich in vegetables and fruits. However, undesirable side effects of the excessive consumption of vegetables and fruits may occur mainly due to the toxicological properties of certain phytochemicals that are present a wide variety of vegetables and fruits. We also realized that our understanding of both the benefits and risks of vegetables and fruits, and their effective ingredients is limited; and we were unable to identify which vegetables and fruits could be responsible for the formation of lithiasis in the stomach in our patient, which appeared to cause the perforation.

The successful treatment of the patient's symptoms may merit attention. First, an emergency surgical intervention with Roux-en-Y anastomosis was selected for the patient, considering the severe infection and sepsis due to perforation of the alimentary canal; although performing this technique for patients who could not tolerate a surgical procedure remains controversial. We consider that the timely removal of the perforation largely contributed to the improvement of the patient's symptoms. Second, the patient developed ICUAW, which is a very common complication of critical illness. This can lead to disability that would persist for years with significantly adverse effects on survivors.^[6]^ Further researches have suggested that ICUAW was closely related to sepsis and systemic inflammatory response syndrome (SIRS).^[7,8]^ Medications for antishock therapy against infection and bedside rehabilitation exhibited effectiveness in the treatment of ICUAW in our patient. Third, anticoagulation therapy using low molecular weight heparin played an important role in restoring blood flow in the patient.^[9]^

In summary, we illustrated an unusual case of perforation of the alimentary canal and other clinical conditions associated with Bigu fasting, raising serious concerns or a warning flag about practicing Bigu in China and other parts of the world. Furthermore, it is strongly advocated that a state of Bigu for a long period of time can even be dangerous.
